# Homologous Recombination Subpathways: A Tangle to Resolve

**DOI:** 10.3389/fgene.2021.723847

**Published:** 2021-08-02

**Authors:** Amira Elbakry, Markus Löbrich

**Affiliations:** Radiation Biology and DNA Repair, Technical University of Darmstadt, Darmstadt, Germany

**Keywords:** homologous recombination, pathway choice, synthesis-dependent strand annealing, ATRX, RECQ5, double-strand break, holliday junction, crossover

## Abstract

Homologous recombination (HR) is an essential pathway for DNA double-strand break (DSB) repair, which can proceed through various subpathways that have distinct elements and genetic outcomes. In this mini-review, we highlight the main features known about HR subpathways operating at DSBs in human cells and the factors regulating subpathway choice. We examine new developments that provide alternative models of subpathway usage in different cell types revise the nature of HR intermediates involved and reassess the frequency of repair outcomes. We discuss the impact of expanding our understanding of HR subpathways and how it can be clinically exploited.

## Repair of DSBs by HR

Cells have evolved multiple mechanisms to preserve genome integrity and restore structural and functional properties of the genome following DNA damage. DNA double-strand breaks (DSBs) are critical lesions whose timely and accurate repair is important for cellular viability and genomic stability. Cells are equipped with multiple pathways to repair DSBs, the most prominent of which are non-homologous end-joining (NHEJ) and homologous recombination (HR). HR provides a high-fidelity mechanism for repair in cycling cells but is restricted to the S and G2 phases of the cell cycle. In contrast to NHEJ, which involves ligating the break ends together, HR involves copying sequences from an intact donor to restore any lost information. HR is also important for the faithful duplication of the genome by providing means of tolerating replication stress and overcoming lesions resulting from replication fork obstruction such as single-stranded DNA (ssDNA) gaps and one-ended DSBs.

Homologous recombination at DSBs can proceed in multiple subpathways, but the initial steps are functionally similar and involve common factors. Briefly, HR commences with the 5ꞌ–3ꞌ extensive resection of break ends by nucleases to generate 3ꞌ ssDNA overhangs, which are then coated by replication protein A (RPA). The breast and ovarian cancer susceptibility protein 2 (BRCA2) then loads the recombinase RAD51 to ssDNA, replacing RPA and forming a nucleoprotein filament to initiate the homology search for complementary sequences. Once homology is found, a displacement loop (D-loop) is formed, where a primer-template junction allows DNA repair synthesis to proceed. After repair synthesis completion, HR can proceed by the displacement of the extended break end from the D-loop and annealing to the complementary sequence at the non-invading end, a subpathway referred to as synthesis-dependent strand annealing (SDSA). An alternative mechanism involves the formation of a joint structure containing a four-way junction between the recombining strands, known as a Holliday junction (HJ). This can occur through the annealing of the non-invading end to the displaced strand of the D-loop in a second-end capture step, or possibly by simultaneous invasion of the two resected ends into the donor and subsequent extension. To allow proper chromosome segregation, the two intertwining strands must be separated, which can occur through two mechanisms with distinct genetic outcomes. Double HJs (dHJs) are prominent HR intermediates and are predominantly processed by helicase- and topoisomerase-dependent dissolution that separates the recombining molecules without genetic exchanges. Alternatively, these joint molecules (JMs, used in this manuscript to refer to post DNA synthesis structures) can be resolved by the structure-selective nucleases to give rise to crossover (CO) or non-CO products at an expected equal frequency. HR can also proceed through a third, non-canonical subpathway termed break-induced replication (BIR), which is characterized by long-range conservative DNA synthesis from the invading DSB end without engagement of the second end and displaying a high propensity to form genomic rearrangements and point mutations. Over the past decade, our understanding of DSB repair pathway choice between NHEJ and HR was greatly enhanced (a topic also reviewed in this issue), which proved useful in many applications, including delineating mechanisms of cellular responses to cancer therapy and finding new drug targets. However, less attention has been paid to HR subpathway choice, our understanding of which falls short especially in human cells. This review aims to focus on the differences between the known HR subpathways, what is known about subpathway choice and the mechanistic and clinical implications of these distinct mechanisms.

## HR Subpathway Outcomes

One feature that is often used to distinguish between the different HR subpathways is their propensity to cause genetic exchanges. Although canonical HR is known to be of high fidelity compared to NHEJ, COs are considered harmful as they can lead to loss of heterozygosity (LOH) if exchanges occur between homologous chromosomes (Moynahan and Jasin, 1997). Translocations, deletions, or inversions can result if COs occur between repeated DNA by non-allelic homologous recombination (Moynahan and Jasin, 1997; Wright et al., 2018). However, it is worth noting that these occur rarely as spontaneous events and while promoted by DSB induction, they are largely suppressed in somatic cells (Stark and Jasin, 2003). An important mechanism to attenuate possible detrimental outcomes is the predominant use of the sister chromatid as donor instead of the homologous chromosome, which renders COs genetically silent (Kadyk and Hartwell, 1992; Soutoglou et al., 2007). Additionally, cells can employ pathways that inherently avoid these products, such as SDSA, which is believed to be the predominant HR subpathway for DSB repair. However, as COs are mostly inconsequential, it is not sufficient to assume cells always favor a CO-avoiding pathway and so the propensity for causing genetic exchanges does not provide an adequate explanation for HR subpathway choice.

While SDSA altogether avoids the formation of HR intermediates that can lead to COs, the processing of such intermediates can also be regulated to favor non-CO products. Consistent with this, dHJs are predominantly dissolved by the BLM-TopoIIIα-RMI1/2 (BTR) complex to non-COs, with CO-prone resolution acting as a last resort to handle these intermediates (Sarbajna and West, 2014). Yet whether dHJs are the only, or even the main, intermediates leading to COs is in question and it remains unclear under which conditions cells favor CO-forming vs. CO-avoiding pathways for DSB repair. Consequently, a more careful dissection of how HR subpathways are regulated and the factors involved are warranted for a better understanding of how distinct repair outcomes arise.

## Revisiting HR Subpathway Choice

In recent years, work by us and others has aimed to define factors involved in promoting and regulating HR subpathway usage. Subpathway choice is often connected to helicases, which can function to either disrupt HR intermediates (such as RAD51 filaments and D-loops), or to promote DNA synthesis and D-loop extension. Therefore, it is important to understand what governs the stability of these intermediates and how they differ in the distinct subpathways. Multiple helicases have been implicated to regulate HR in human cells, including RTEL (Barber et al., 2008), BLM (van Brabant et al., 2000), FANCJ (Sommers et al., 2009), FBH1 (Chan et al., 2018), RECQ1 (Bugreev et al., 2008) and RECQ5 (Hu et al., 2007; reviewed in Huselid and Bunting, 2020). Helicases that disrupt the pre-synaptic RAD51 filaments by enhancing RAD51 removal are referred to as anti-recombinases due to their HR-limiting functions. Conversely, disruption of extended D-loops serves to limit the extent of DNA synthesis and displace the invading strand to channel repair toward SDSA. Often, helicases harbor both anti- and pro-recombinogenic biochemical functions, making it difficult to pinpoint the precise contribution of these helicases to DSB repair. Recently, we have found that at two-ended DSBs, RECQ5 promotes a repair pathway, likely to be SDSA, involving short-range repair synthesis and resulting in non-CO repair products. The role of RECQ5 in this context is unclear, although it has been implicated to involve RAD51 removal after strand displacement to prevent re-invasion cycles and allow strand annealing to promote SDSA (Paliwal et al., 2014). Other functions could relate to those of analogous yeast helicases, such as Srs2, that involve disrupting D-loops and limiting DNA synthesis (Burkovics et al., 2013; Liu et al., 2017). Additionally, some reports support a requirement for only the helicase domain of Srs2 for its SDSA function (Bronstein et al., 2018), and others find that also the RAD51-interacting domain has an effect on CO formation (Jenkins et al., 2019), rendering the precise role of the helicase uncertain. Furthermore, factors regulating strand annealing post displacement are not well-defined, although, differential processing of the non-invading break ends has been implicated. Successful engagement of the second end is important to terminate repair of two-ended breaks and its failure can activate one-ended DSB repair mechanisms, such as BIR (Chandramouly et al., 2013). Consistently, the length of homology between the non-invading end and the displaced strand, influenced by the extent of resection, has been suggested to regulate subpathway choice between SDSA and BIR in human cells. Similarly, asynchronous resection and short homology tracts lead to failure of strand annealing and activation of BIR in yeast, a process regulated by the Mph1 and Sgs1 helicases, which dismantle D-loops (to favor SDSA) or short homology duplexes (to promote BIR), respectively (Mehta et al., 2017; Pham et al., 2021).

Alternatively, HR can proceed through a RAD54-dependent pathway with a propensity for CO formation (Spies et al., 2016). We have shown that the chromatin remodeler ATRX promotes this subpathway of HR that involves long stretches of DNA synthesis leading to the formation of a high frequency of COs visualized as sister-chromatid exchanges (SCEs; Juhász et al., 2018). Interestingly, the HR intermediates formed by this pathway, which can be detected as IR-induced ultra-fine bridges (UFBs), are completely dependent on the structure-selective nucleases MUS81 and GEN1 for resolution and are independent of BLM function (Elbakry et al., 2021). Since BLM has a well-documented role in suppressing endogenous SCEs, HR at two-ended DSBs can lead to distinct structures than those formed at replication-associated lesions that may not be classic dHJ and are therefore processed differently. This is consistent with studies reporting high CO levels and synthetic lethality of cells lacking GEN1 and SLX4 (essential for MUS81 function at HJs) even in the presence of BLM, indicating the presence of HJs that exclusively require resolution (Garner et al., 2013; Wyatt et al., 2013). Thus, it appears that one subpathway of HR DSB repair strictly forms a type of JM that requires resolution, the mechanism of which remains unclear (discussed below).

**Figure 1:**
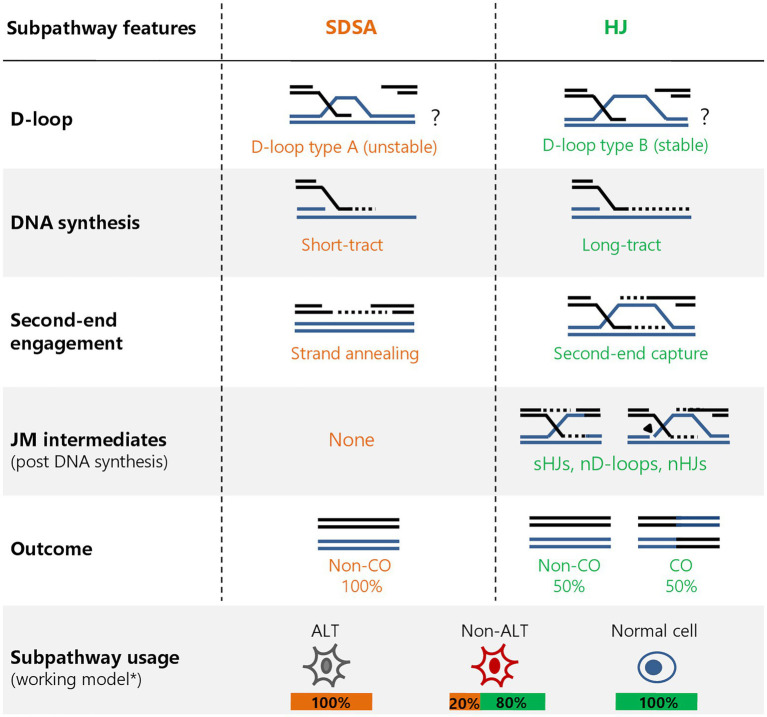
Homologous recombination (HR) subpathway features at two-ended DNA double-strand breaks (DSBs). HR at two-ended breaks can proceed in distinct subpathways after RAD51-mediated strand invasion: synthesis-dependent strand annealing (SDSA; orange) and the Holliday junction (HJ; green) subpathway. Distinguishing features include the type and stability of displacement loop (D-loop; based on yeast models), extent of DNA repair synthesis, mode of second-end involvement, formation of joint molecules (JMs) post DNA synthesis (including single or nicked HJs and nicked D-loops) and repair outcome [crossover (CO) or non-CO]. Different types of cells use the subpathways to varying extents, largely influenced by their alternative lengthening of telomeres (ALT) status (see this paper). ^*^These frequencies are non-comprehensive and are subject to change as more cell lines are analyzed.

Strikingly, cells lacking ATRX expression, such as U2OS cells, rely completely on RECQ5 for HR-mediated repair of DSBs but are able to switch to the ATRX subpathway upon the induction of ATRX expression (Elbakry et al., 2021). The regulation of pathway choice seems to be dependent on proliferating cell nuclear antigen (PCNA) interaction, as both ATRX and RECQ5 possess PCNA-interaction peptide (PIP) domains that are essential for their HR function. Repair studies using mutants suggest that ATRX and RECQ5 compete for PCNA binding, possibly involving post-translational modifications (PTMs) that could regulate the downstream processes (Elbakry et al., 2021). The possibility of PTM-mediated regulation would be consistent with a role of RECQ5-dependent PCNA ubiquitination as well as PCNA-SUMO2 conjugation during transcription-replication conflict resolution, which serve to remove PCNA and RNA polymerase II from chromatin, respectively (Urban et al., 2016; Li et al., 2018). Additionally, it has been shown that yeast Srs2 interacts with SUMO-PCNA to promote SDSA by regulating the DNA polymerase, or by dissociating heteroduplex DNA (hDNA) at the D-loop and allowing second-end annealing and repair completion (Burkovics et al., 2013; Liu et al., 2017). Whether these PTMs or others influence HR outcome remains to be determined and would provide valuable insights about the regulation of subpathway choice during HR. This is particularly relevant considering that different cell types utilize the subpathways to various extents. For example, while ATRX-deficient cancer cells seem to rely on RECQ5 for HR, normal untransformed cells do not use RECQ5 and rely completely on ATRX for HR (Elbakry et al., 2021). Conversely, ATRX-proficient cancer cells, like HeLa cells, exhibit an uneven contribution from the two subpathways (Figure 1). This discrepancy in HR subpathway usage warrants a re-examination of a general one-size-fits-all model for the repair of breaks *via* HR and demands a more careful attention to the model systems and cell lines used. Differential subpathway usage also provides a novel way to assess HR proficiency in cancer cells that have a particular reliance on one subpathway or the other. Therefore, instead of solely focusing on upstream factors like BRCA1/2 and RAD51, we should also consider the downstream processes that define which subpathways are operating in the cell.

## HR Subpathways: Making Ends Meet

Understanding how HR subpathways function and how the choice between them is regulated requires the consideration of the repair outcomes these pathways produce. The preference of a pathway favoring the formation of SCEs to one that avoids them challenges the common dogma that cells inherently avoid COs at all costs. To explain this discrepancy, it is worth examining how these conclusions were established. Many of the studies reporting rare CO occurrence were carried out using HR reporters in mammalian cells, and while they highly contribute to understanding pathway choice, the limitations of these systems could mask or skew these frequencies. One such limitation is reliance on ectopic or integrated artificial constructs that detect unequal recombination events or recombination between homologous chromosomes, all of which do not reflect the natural HR substrate of the identical sister (Johnson and Jasin, 2000; LaRocque et al., 2011; Zapotoczny and Sekelsky, 2017). Indeed, CO frequencies close to 50% can be observed in yeast, where the homologous chromosome represents a more natural recombination substrate (Ho et al., 2010; Yim et al., 2014). Furthermore, genetic analysis of the products in reporter systems in mammalian cells often differentiates only between long-tract gene conversion (LTGC) events and COs arising from short-tract gene conversion (STGC) and do not take into consideration that CO events arising from longer DNA synthesis tracts would be indistinguishable from non-CO LTGC events (Johnson and Jasin, 2000). This likely underestimates the frequency of CO in these systems, since COs have been associated with pathways involving longer tracts of DNA synthesis (Elliott et al., 1998; Mitchel et al., 2010; Yim et al., 2014). Additionally, the genetic background of cells used, such as U2OS cells that lack an HR factor, should be considered as this also affects the results from these reporters (Juhász et al., 2018; Elbakry et al., 2021). Therefore, while the notion that CO-avoiding pathways are preferred may or may not be valid, it is imperative to consider other factors influencing pathway choice and repair outcome. Cells deal with the various DNA-damaging lesions in distinct mechanisms, and those arising during S phase, which give rise to the majority of the spontaneous SCEs, could be handled differently from those at two-ended DSBs. As has been observed in yeast, recombination at ssDNA gaps results in dHJs requiring dissolution by Sgs1, while DSB-generated structures rely on nuclease-mediated resolution (Ho et al., 2010; Giannattasio et al., 2014). Therefore, the structure and nature of the lesion could influence the HR intermediates formed and how they are processed and consequently, whether or not they lead to genetic exchanges. Similarly, the genomic location of the lesion can dictate outcome, as has been shown for DSB repair pathway choice between HR and NHEJ (Beucher et al., 2009; Goodarzi et al., 2010; Aymard et al., 2014). Consistent with this, locus-specific SCE analysis showed that early and late replicating fragile sites exhibit differential SCE frequencies, suggesting that genomic locus and chromatin architecture could also influence HR subpathway choice (Waisertreiger et al., 2020). Furthermore, recent studies have demonstrated distinct mechanisms of HR-mediated repair of DSBs occurring in transcribed regions vs. transcriptionally silent loci, implicating the formation of DNA:RNA hybrids as novel regulators of HR (Yasuhara et al., 2018; Ouyang et al., 2021).

Notwithstanding the underlying mechanism, the preference of CO-forming pathways in certain contexts suggests that this is probably a less toxic outcome than products from alternative pathways. It is not yet clear how this could be the case for SDSA vs. a pathway involving a more complicated HR intermediate joining the two chromatids, as the fidelity of either subpathway has not been closely examined in the specific context of two-ended breaks. It is possible that factors such as D-loop stability, polymerase choice, and the fidelity of second-end engagement may play a role in ensuring accurate repair, even at the cost of an increased risk of CO formation. Not much is known about the regulation of the annealing step during SDSA and how the cell ensures the involvement of the correct ends. As HR normally deals with endogenous breaks that arise at replication forks that have one end, employing pathways that require two ends dictates the need to “wait” until a second end is generated by an approaching replication fork. In this context, the premature displacement of the invading strand could cause its annealing to a non-matching break end, leading to translocations, a more detrimental outcome than a genetically silent CO (Ensminger and Löbrich, 2020). Therefore, a subpathway that has a more stringent second-end annealing condition, like second-end capture by the displaced D-loop strand, could be preferred. Second-end capture ensures enough homology is met, and also involves a structure refractory to termination by other pathways, like end-joining and/or single-strand annealing.

Furthermore, one pathway may involve a more stable intermediate structure that is resistant to dissociation and thereby could be favored to ensure repair completion. For example, studies utilizing novel D-loop analysis assays in yeast have shown the formation of two distinct D-loop species, whose lengths and abundance are regulated by Rad54 and its paralog Rdh54/Tid1 (Piazza et al., 2019; Shah et al., 2020). The features of the different D-loop species make them resistant to specific helicases and alterations in these properties influence HR outcome and survival. It is tempting to speculate that a similar mechanism may occur in human cells and can dictate subpathway choice (Figure 1). In this context, ATRX may cooperate with human RAD54 to form more stable intermediates (Spies et al., 2016; Juhász et al., 2018), possibly through a mechanism involving histone deposition within the D-loop (Elbakry et al., 2018). To investigate these possibilities, the required tools (e.g., D-loop capture and extension assays) need to be adapted and optimized for human cells. Furthermore, D-loop stability and extension can also be promoted by RNA:DNA hybrids arising during HR at transcribed regions (forming DR-loops), a feature that could influence subpathway choice (Ouyang et al., 2021).

While multiple factors can skew HR outcome by influencing subpathway usage, frequent COs during DSB repair can arise during the processing of HR intermediates that are preferentially channeled toward resolution instead of the CO-avoiding dissolution (Elbakry et al., 2021). This is a scenario where the structure-selective nucleases represent the main, rather than the back-up, pathway to handle JMs. Although, the activation of the MUS81-SLX4 and GEN1 complexes during late G2/M phase of the cell cycle (Pfander and Matos, 2017) could explain the preferential use of the nucleases at this stage, it does not exclude a role for the BTR dissolvasome, and raises the question if these JMs are intact dHJs, or in fact, dHJs at all (Figure 1). The preferential formation of COs from HR junctions has been indicated by the analysis of hDNA tracts in yeast and suggested a bias toward resolution explained by the presence of nicked or single HJs, which are more suitable substrates to the nucleases (Mitchel et al., 2010). Additionally, the formation of anaphase bridges arising from non-canonical HJs has been observed in yeast and was found to be specific to resolvase-deficient cells (García-Luis and Machín, 2014). Therefore, alternative JMs that are distinct from the canonical dHJ have been proposed in various contexts of HR by us and others and potentially occur more frequently than previously thought (Wright et al., 2018; Machín, 2020; Elbakry et al., 2021). The presence and frequency of these structures is yet to be determined and would both reflect the usage of distinct subpathways and dictate the requirement of specific downstream processing factors. Therefore, we find the use of the more general term “HJ pathway” more accurate when dealing with pathways involving JMs in DSB repair.

## Clinical Implications of HR Subpathway Choice

**Figure 2:**
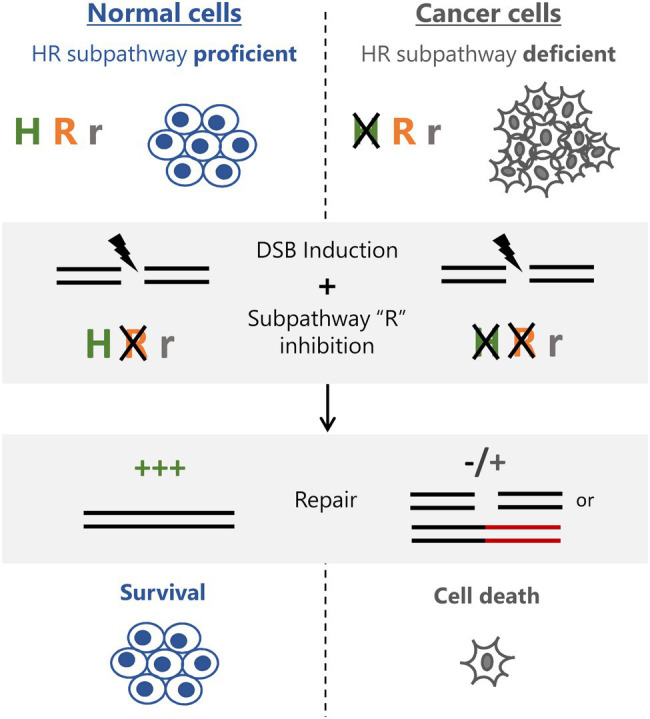
Exploiting HR subpathway usage for cancer therapy. Normal cells have intact homologous recombination repair (HRR) and use predominantly subpathway “H” for DSB repair. Cancer cells lacking subpathway “H” rely predominantly on subpathway R for repair. Inhibiting subpathway “R” does not affect normal cells, which can repair normally and survive treatment. Cancer cells lose their main HRR functionality and either fail to repair or depend on more erroneous pathways (subpathway “r”) leading to accumulation of breaks and/or toxic lesions and subsequent cell death.

Homologous recombination deficiency has been used to target cancer cells for therapy ever since the concept of synthetic lethality has been elegantly shown in BRCA1/2 deficient cells treated with PARP inhibitors (Bryant et al., 2005; Farmer et al., 2005). This success has fueled further studies to identify other synthetically lethal targets in BRCA-defective cells, as well as cells deficient in other HR factors. Therefore, with an even deeper understanding of HR and the different subpathways involved, new strategies can be employed to effectively kill cancer cells. For example, cells that are defective in canonical HR subpathways and over-rely on other subpathways, such as BIR, can be selected by targeting BIR-specific factors. Alternatively, tumors lacking factors involved in particular subpathways can be targeted by identifying new synthetic lethal interactions specific to these tumors (Figure 2). Further, as demonstrated recently, the loss of the BIR factor PIF-1 can be exploited for selective killing of cells made to rely on this HR subpathway by the concurrent deletion of FANCM, revealing a new synthetic lethality relationship and an approach to target PIF-1 mutant cancer cells (Li et al., 2021). Also, it is known that the HR factor ATRX is defective in a variety of tumors that are commonly using the alternative lengthening of telomeres (ALT) mechanism of telomere maintenance (representing around 10–15% of all cancers; Dilley and Greenberg, 2015). While it is still not completely clear how loss of ATRX contributes to the ALT phenotype, exploiting a possible HR pathway imbalance (i.e., higher dependence on SDSA in tumors lacking ATRX), regardless of ALT status, could prove an effective approach to target these cells (Figure 2). This is particularly attractive if, as demonstrated, normal cells rely on the ATRX pathway for repair. Therefore, as the interplay between the HR subpathways becomes clearer and more defined, the therapeutic window of exploiting HR subpathways will expand, justifying a need for a better understanding of the mechanisms governing pathway choice.

## Author Contributions

AE and ML designed, revised, and edited the manuscript. AE wrote the first draft. All authors contributed to the article and approved the submitted version.

## Conflict of Interest

The authors declare that the research was conducted in the absence of any commercial or financial relationships that could be construed as a potential conflict of interest.

## Publisher’s Note

All claims expressed in this article are solely those of the authors and do not necessarily represent those of their affiliated organizations, or those of the publisher, the editors and the reviewers. Any product that may be evaluated in this article, or claim that may be made by its manufacturer, is not guaranteed or endorsed by the publisher.
